# The effects of sodium hydrogen carbonate ingestion during the recovery period between two 200-m front-crawl time trials

**DOI:** 10.1007/s00421-024-05522-2

**Published:** 2024-06-06

**Authors:** Anton Ušaj, Robert Marčun, Boro Štrumbelj

**Affiliations:** 1 Faculty of Sport, Laboratory of Biodynamics, University of Ljubljana, Ljubljana, Slovenia; 2 University Clinic of Pulmonary and Allergic Diseases Golnik, University of Ljubljana, Golnik, Slovenia

**Keywords:** Blood alkalosis, Acidosis, Blood gases, Acid–base balance

## Abstract

**Purpose:**

The aim of this study was to determine how sodium hydrogen carbonate (NaHCO_3_) ingestion during a 1-h recovery period after a 200-m front-crawl swim affects blood–gas levels, acid–base balance, and performance during a successive trial.

**Methods:**

Fourteen national-level male swimmers (age: 21 ± 3 years, body mass (BM):77 ± 10 kg, stature: 181 ± 7 cm) performed four maximal 200-m front-crawl tests. On one of the two days, the swimmers swam two 200-m tests with a 1-h recovery break, during which they drank water (WATER); on the other day, they performed the same protocol but consumed 0.3 g min^−1^ NaHCO_3_ solution during the recovery break (NaHCO_3_).

**Results:**

The ingestion of NaHCO_3_ before the second test had no effect on swim time despite a greater [${HCO}_{3}^{-}$] (19.2 ± 2.3 mmol L^−1^) than that measured during the first test (NaHCO_3_) (14.5 ± 1.1 mmol L^−1^) and the other two tests (WATER) (12.7 ± 2.4 and 14.8 ± 1.5 mmol L^−1^; *F* = 18.554; *p* = 0.000) and a higher blood pH (7.46 ± 0.03) than that measured during the first test (NaHCO_3_) (7.39 ± 0.02) and the other two tests (WATER) (7.16 ± 0.04 and 7.20 ± 0.05); (*F* = 5.255; *p* = 0.004). An increase in blood pCO_2_ (0.2 ± 0.3 kPa) between both tests (NaHCO_3_) compared to unchanged pCO_2_ values (− 0.1 ± 0.3 kPa) between the other two tests (WATER) (*t* = − 2.984; *p* = 0.011; power = 0.741) was confirmed.

**Conclusions:**

NaHCO_3_ ingestion during the recovery period between two 200-m front-crawl time trials had a strong buffering effect that did not positively affect performance. An increase in pCO_2_ may have counterbalanced this impact.

## Introduction

International swimming competitions for swimmers contesting the 200-m front-crawl event consist of a relatively long, multiday programme or at least a 1-day programme with two events: a qualification trail or a semifinal and a final trial. The recovery interval between two trials ranges from several hours to several days. However, if someone competes in more than one swimming discipline, which usually occurs at national-level competitions, the recovery interval may decrease to 1 h. It is assumed that this time interval is not sufficient for complete recovery if passive recovery is used. Therefore, different recovery strategies have been tested in the past to improve recovery (Lomax 2012; Toubekis et al. 2005). Anecdotal information from swimmers shows that some swimmers utilise passive recovery, other swimmers utilise active recovery through low-intensity swimming, and other swimmers utilise active recovery through the ingestion of sodium hydrogen carbonate (NaHCO_3_). There is a long tradition of research on the potential effects of NaHCO_3_ ingestion on sport performance (McNaughton et al. 2008; Carr et al. 2011; Shelton and Kumar 2010) and specifically on swimming performance; however, the results are equivocal. Some studies support the idea that NaHCO_3_ ingestion affects swimming performance during the front-crawl stroke (Lindh et al. 2007; Siegler et al. 2010; Mero et al. 2013), while others do not (Joyce et al. 2012). Moreover, several studies in other sports (Driller et al. 2013; Stephens et al. 2002; Katz et al. 1984; Price and Simons 2010) did not report significant effects of NaHCO_3_ ingestion on performance. Because of differences in research designs and inconclusive results, to date, it is not possible to predict whether NaHCO_3_ ingestion during the 1-h recovery period between swims is an adequate intervention against fatigue, allowing the swimmer to be maximally prepared for the successive swim. Therefore, the first aim of this study was to determine whether NaHCO_3_ ingestion during the 1-h recovery period between two 200-m front-crawl time trials significantly affects swimming performance in the second trial.

When determining the effects of NaHCO_3_ ingestion during swimming, two observations should be considered: (a) the complex effects of blood ${\text{HCO}}_{3}^{-}$ buffering (Siggaard–Andersen 1976; Stewart 1981; Robergs et al. 2004) and (b) the fact that breathing is restricted during front-crawl swimming (Cardelli et al. 2000; Ušaj 1999; Kapus et al. 2008; Couto et al. 2014). The complex function of the ${\text{HCO}}_{3}^{-}$ buffer system in the blood during exercise involves the exchange of electrolytes and water between blood and muscles (Lindinger et al. 1999; Sejersted and Sjøgaard 2000; Clausen 2003). Moreover, increased metabolism leads to the accumulation of muscle lactate (LA^−^) and hydrogen ions (H^+^) (Robergs et al. 2004). This complex rearrangement of the intracellular and transmembrane ion concentrations changes the ion balance and the intracellular strong ion difference (SID) (Stewart 1981; Jones 1990). The increase in intracellular CO_2_ due to aerobic processes and as a product of intracellular H^+^ buffering decreased the intramuscular pH. Due to the energy-consuming opposite transport of K^+^ and Na^+^ (Sejersted and Sjøgaard 2000; Clausen 2003), the active cotransport of LA^−^ and H^+^ and facilitated CO_2_ transport from intracellular to extracellular compartments and the blood, the ion balance of the blood, represented by an anion gap (Siggaard-Anderson 1976) or a SID (Stewart 1981), changes towards blood acidosis and hypercapnia. The large blood volume and ion transport across the muscle membrane partially compensates for muscle electrolyte changes in the muscles, while the changes in the acid–base balance are strongly compensated for by the ${\text{HCO}}_{3}^{-}$ buffer system in the blood. The effective association between H^+^ and ${\text{HCO}}_{3}^{-}$ to form H_2_CO_3_ by the enzyme carboanhydrase (CA) and their dissociation to CO_2_ and H_2_O effectively regulates blood acidosis when CO_2_ can be successfully released from the blood into the air by intensive respiration (respiratory compensation of metabolic acidosis) (Siggaard-Anderson 1976, Astrand et al. 2003). As mentioned above, the front-crawl event in particular is an exercise in which a limited increase in ventilation is likely to reduce the power of this final stage of the ${\text{HCO}}_{3}^{-}$ buffer system. Consequently, blood pCO_2_ may not decrease below resting levels, as in running (unpublished results) and kayaking (Ušaj 1999). Whether performance is affected by this phenomenon is not clear (Graham et al. 1980). With increasing blood alkalosis and [${\text{HCO}}_{3}^{-}$] influenced by pre-exercise NaHCO_3_ ingestion, the excess CO_2_ that accumulates from aerobic metabolism and from ${\text{HCO}}_{3}^{-}$ buffering of H^+^ must be exhaled as excess CO_2_ by additional increased ventilation. However, this may not be sufficient to prevent hypercapnia during maximal-effort front-crawl swimming, as ventilation is limited. The second aim of the study was to determine the possible dual and opposing influence of NaHCO_3_ ingestion, by which it could result in enhanced front-crawl performance due to increased ${\text{HCO}}_{3}^{-}$ buffering capacity (Hollidge-Horvat et al. 2000; Street et al. 2005; Lindinger et al. 1989; Raymer et al. 2004; Sostaric et al. 2006) or decreased performance due to the limited increase in ventilation and increased hypercapnia.

## Methods

The research design was required that front–crawl tests were performed with maximal intensity. Whether the swimmers’ performance during the 200-m tests was actually close to their expected maximum potential at that time was tested for 8 out of 14 swimmers by comparing the official 200-m front-crawl competition results of the already completed competition season with the results of their first 200-m test during the study using a paired-samples t test. If during the first 200 m test, the swimmers performance corresponded to their expected maximum potential for that period of time, and in further analyses, this correspondence was logically assumed to be similar for the whole group of 14 swimmers. This was done because the other swimmers had finished their competition season earlier, and their competition results could not be used to estimate whether they swam at their expected maximum potential, i.e. similar to their official competition performance.

A group of 14 national and international swimmers (200-m front-crawl time, 123.24 ± 5.94 s; FINA World Aquatics swimming points for 25 m swimming pools, 529.87 ± 72.03; age, 21 ± 3 years; body mass (BM), 77 ± 10 kg; stature, 181 ± 7 cm; body mass index (BMI), 23 ± 2) participated in the study. All of them had at least 4 years of experience in 100- and 200-m front-crawl competitions. At the time of the testing, they had already completed their competitive season. All swimmers were verbally informed about the aims of the study and the potential risks and challenges associated with the experimental procedures. All swimmers provided written informed consent. The study was approved by the National Committee for Medical Ethics at the Ministry of Health of the Republic of Slovenia (0120-159/2023/3) and was conducted in accordance with the current guidelines of the Declaration of Helsinki.

**Fig. 1:**
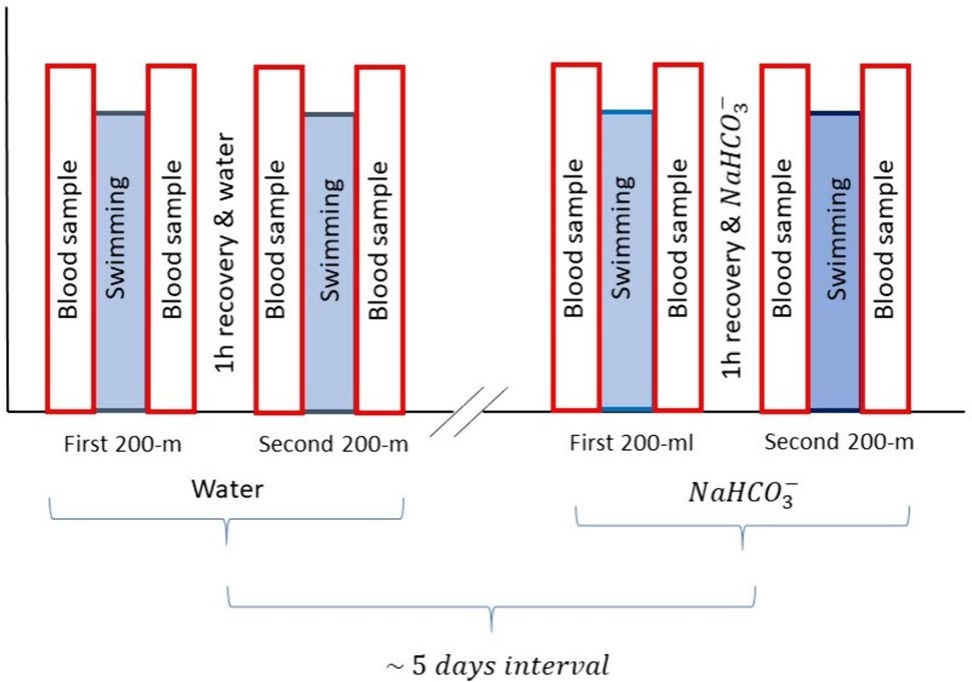
Design of the experiment. The experiment consisted of two drinking solution conditions. The WATER condition consisted of two 200-m tests separated by a 1-h recovery break with water ingestion. Blood samples were taken before and after each trial. Another condition (NaHCO_3_) consisted of two 200-m tests, separated by a 1-h recovery break with the ingestion of NaHCO_3_ solution. As with the WATER, blood samples were taken before and after each 200-m test

All swimmers individually completed four maximal-effort 200-m swim tests: two as water condition tests (WATER) on one simulated competition day and two as NaHCO_3_ condition test (NaHCO_3_) on another simulated competition day; the two conditions tests were approximately 5 days apart (Fig. 1). The participants were instructed to breathe on every second stroke while swimming. The times for the 200-m front-crawl trials were measured by an experienced swimming coach using a Seiko stopwatch S141-300 Lap Memory Stopwatch (Japan). The times were analysed with an accuracy of ± 0.1 s, while the competition results were measured with an official electronic system with an accuracy of ± 0.01 s. Nevertheless, their comparison was performed with an accuracy of ± 0.1 s. The stroke rate was calculated using the 3-cycle base function on the stopwatch. The stopwatch was started as soon as the swimmer’s hand entered the water to begin a stroke. After three complete strokes, the stopwatch was stopped when the same hand entered the water for the fourth stroke. A standard warm-up routine was performed before the first trial, followed by an individual maximum 200-m front-crawl trial. The trials were filmed, and the breathing rate was checked to ensure that the prescribed breathing rate was maintained. Everyone was encouraged to swim at maximum effort. This attempt was followed by an hour of recovery, a warm-up and a second 200-m trial.

Approximately, 5 min before each start, each swimmer's earlobe was moistened with Capsolin (Laboratorio Farmaceutico SIT, Mede, Italy) cream, which has a strong hyperaemic effect. Approximately, 90 μl of arterialised capillary blood was collected in Clinitubes (D941 G-240-85 capillary tubes; Radiometer, Copenhagen). The thin steel wire was inserted into the tube, and the end points were sealed with wax. The blood sample was carefully mixed by moving a small magnet along the capillary tube and was then stored on ice (Haskins 1977). During the first minute of the recovery period, the second 90 μl of capillary blood was collected and prepared in the same way as that collected before the swim test. Blood gas and electrolyte levels were measured within 1 h using an ABL800FLEX (Radiometer, Copenhagen) blood gas and electrolyte analyser. The analyser was automatically calibrated at regular intervals according to the manufacturer’s instructions.

The measurement of the swimmers’ heart rate started 5 min before the warm-up and continued during the 200-m swim and during the 1-h recovery period during the second 200-m trial and the final 5-min recovery. A Polar OH1 optical sensor (Polar, Finland) was attached to the temple of the head and was wirelessly connected to an iPad (Apple, USA).

On a randomly selected test day, a solution of 0.3 g∙kg^−1^ NaHCO_3_ dissolved in 0.5 L of water was ingested during the first 30 min of the 1-h recovery period (NaHCO_3_). On the other test day, the swimmers drank water, which was flavoured in a similar way to the NaHCO_3_ solution (WATER). Nevertheless, most swimmers correctly recognised the taste of the NaHCO_3_ solution, either due to previous experience consuming NaHCO_3_ or due to a change in digestion before the second trial. Therefore, the design was not double-blinded.

Four data sets collected during the first and second WATER 200-m tests and during the first and second NaHCO_3_ 200-m tests (Fig. 1) were first tested for normality using the Shapiro–Wilk test. The data sets were then compared for differences in front-crawl times, blood acid–base parameters (pH, pCO_2_, [${\text{HCO}}_{3}^{-}$], base excess (BE) and [LA]), blood oxygen saturation (SaO_2_) and the partial pressure of O_2_ at which SaO_2_ reached 50% (P_50_). The parameters were compared by repeated-measures analysis of covariance (ANCOVA), where values of the first reference trial were used as covariates, and Bonferroni correction was used for multiple comparisons. The homogeneity of variance (sphericity) was analysed using Mauchly tests. If the significance value is > 0.05, the assumption of sphericity is accepted. Partial eta squared values (η^2^) were used to estimate the size of the main effects and were interpreted as follows: trivial (≤ 0.2), small (0.2–0.49), moderate (0.5–0.7) or large (≥ 0.8).

Additional focus was placed on the pCO_2_ changes. In the first analysis, the post-trial pCO_2_ values of the two WATER tests were used, and the difference between these values (∆pCO_2WATER_) was calculated. Similarly, the differences in post-pCO_2_ values between the two NaHCO_3_ tests were also calculated (∆pCO_2NaHCO3_). Finally, paired-samples t tests were used to identify major differences between the two ∆pCO_2_ values.

Perceived exertion was assessed using the 10-level Borg scale (Borg 1982), which ranges from very light exercise (4) to very heavy exercise (24). All swimmers were familiar with this way of assessing perceived exertion, as they used this type of assessment in their training process (Borg 1982).

The data are presented as the means ± SDs unless otherwise stated. *p* ≤ 0.05 was considered to indicate statistical significance, and the statistical analyses were performed using SPSS version 22 software (Chicago, IL, USA).

## Results

The official 200-m front-crawl competition results of the group of eight swimmers who had already completed competition season (123.24 ± 5.94 s) were similar to the results of these swimmers first 200-m test during the study (124.6 ± 5.1 s), measured with a stopwatch (*t* = − 1.29, *p* = 0.24, power = 0.10). The average front-crawl times of the entire group during the four 200-m tests were following: 129.9 ± 10.3 for the first, 131.1 ± 9.7 for the second test with water consumption, 131.0 ± 10.4 for the first and 132.3 ± 9.9 s for the second test with NaHCO_3_ consumption, and there were no significant differences in swim time among the four trials (*F* = 0.988, *p* = 0.34, *η*^2^ = 0.123) (Table 1).

**Table 1:** Basic characteristics of the 200-m swimming tests

	WATER		NaHCO_3_	
	First 200 m	Second 200 m	First 200 m	Second 200 m_NaHCO3_
Time 200-m (s)	129.9 ± 10.3	131.1 ± 9.7	131.0 ± 10.4	132.3 ± 9.9
Stroke rate (min^−1^)	40.5 ± 4.8	40.8 ± 5.0	41.3 ± 5.2	41.7 ± 5.3

WATER—denotes water-drinking conditions

NaHCO_3_—denotes NaHCO_3_-drinking conditions

The ingestion of NaHCO_3_ during the 1-h recovery period before the second front-crawl test had strong effects on the acid–base balance of the blood (Table 2). Compared to that during the other three 200-m tests, during the both 200-m tests with water ingestion and the first 200-m test with NaHCO_3_ ingestion, blood pCO_2_ pre only tended to increase by approximately 0.3 kPa (*F* = 1.300; *p* = 0.289; *η*^2^ = 0.088) (Table 2) during second 200-m test with NaHCO_3_ ingestion. However, when comparing ∆pCO_2WATER_ to ∆pCO_2NaHCO3_, there was a significant difference between both drinking conditions (*t* = − 2.251; *p* = 0.043; power = 0.45) (Fig. 3a). Compared to the pretest resting values with water ingestion, blood pH pre increased by approximately 0.05 (*F* = 5.297; *p* = 0.004; *η*^2^ = 0.306) and blood [${\text{HCO}}_{3}^{-}$] pre levels increased by approximately 4.7 mmol∙L^−1^ (*F* = 5.264; *p* = 0.04; *η*^2^ = 0.305) during the recovery interval with NaHCO_3_ ingestion (Table 2). Similarly, compared to the pretest values with water ingestion, blood BE pre increased by approximately 5.4 mmol∙L-^1^ with NaHCO_3_ ingestion (*F* = 14.245; *p* = 0.000; *η*^2^ = 0.543) (Table 2). Blood [LA] pre levels were higher before the second 200-m test with water ingestion and before the second 200-m test with NaHCO_3_ ingestion than before other two first 200-m tests (*F* = 8.476; *p* = 0.000; *η*^2^ = 0.414) (Table 2). These values were probably in excess because of the effects of the previous 200-m test. NaHCO_3_ ingestion had no effect on SaO_2_ pre or heart rate (Table 3). However, NaHCO_3_ ingestion was associated with a decrease in P_50_ pre to 2.8 ± 0.3 kPa, which was lower than that in the other 200-m test with water ingestion (*F* = 4.426; *p* = 0.010; *η*^2^ = 0.287) (Table 3).

**Table 2:** Blood gas, acid–base, and lactate values before and after the 200-m swimming tests

	WATER		NaHCO_3_	
	First 200 m	Second 200 m	First 200 m	Second 200 m
pCO_2_ pre (kPa)	5.0 ± 0.3	4.9 ± 0.4	5.1 ± 0.2	5.4 ± 0.4
pCO_2_ post (kPa)	5.0 ± 0.3	5.0 ± 0.4	5.1 ± 0.5	5.3 ± 0.4
pH pre	7.40 ± 0.03	7.40 ± 0.02	7.39 ± 0.02	7.46 ± 0.03*
pH post	7.16 ± 0.04	7.20 ± 0.05	7.19 ± 0.04	7.30 ± 0.05*
[${HCO}_{3}^{-}$]pre (mmol L^−1^)	23.7 ± 1.4	23.3 ± 1.7	23.7 ± 0.8	28.4 ± 2.4*
[${HCO}_{3}^{-}$]post (mmol L^−1^)	12.7 ± 2.4	14.8 ± 1.5	14.5 ± 1.1	19.2 ± 2.3*
BE pre	− 1.2 ± 1.7	− 1.6 ± 2.2	− 0.9 ± 1.0	4.5 ± 2.8*
BE post	− 15.0 ± 4.4	− 12.1 ± 2.0	− 12.4 ± 1.7	− 6.1 ± 2.9*
[LA]pre (mmol L^−1^)	1.8 ± 0.8	2.3 ± 0.9	1.7 ± 0.4	2.1 ± 0.5*
[LA]post (mmol L^−1^)	14.7 ± 2.3	11.1 ± 2.5	13.3 ± 2.4	13.1 ± 3.5

^*^ *p* < 0.05, different from other trials

WATER—denotes water-drinking conditions

NaHCO_3_—denotes NaHCO_3_-drinking conditions

**Table 3:** Heart rate, blood oxygen saturation and blood oxygen pressure at 50% oxygen saturation in the 200-m swimming tests

	WATER		NaHCO_3_	
	First 200 m	Second 200 m	First 200 m	Second 200 m_NaHCO3_
HR pre (min^−1^)	112 ± 17	116 ± 18	111 ± 11	113 ± 17
HR post (min^−1^)	174 ± 12	178 ± 9	171 ± 15	174 ± 13
SaO_2_ pre (%)	97 ± 1	96 ± 1	97 ± 1	95 ± 2
SaO_2_ post (%)	96 ± 1	96 ± 1	95 ± 2	96 ± 2
P_50_ pre (kPa)	3.3 ± 0.4	3.1 ± 0.4	3.3 ± 0.4	2.8 ± 0.3
P_50_ post (kPa)	4.0 ± 0.5	3.8 ± 0.2	3.7 ± 0.3	3.4 ± 0.4*

^*^ *p* < 0.05, different from other trials

WATER—denotes water-drinking conditions

NaHCO_3_—denotes NaHCO_3_-drinking conditions

**Fig. 2:**
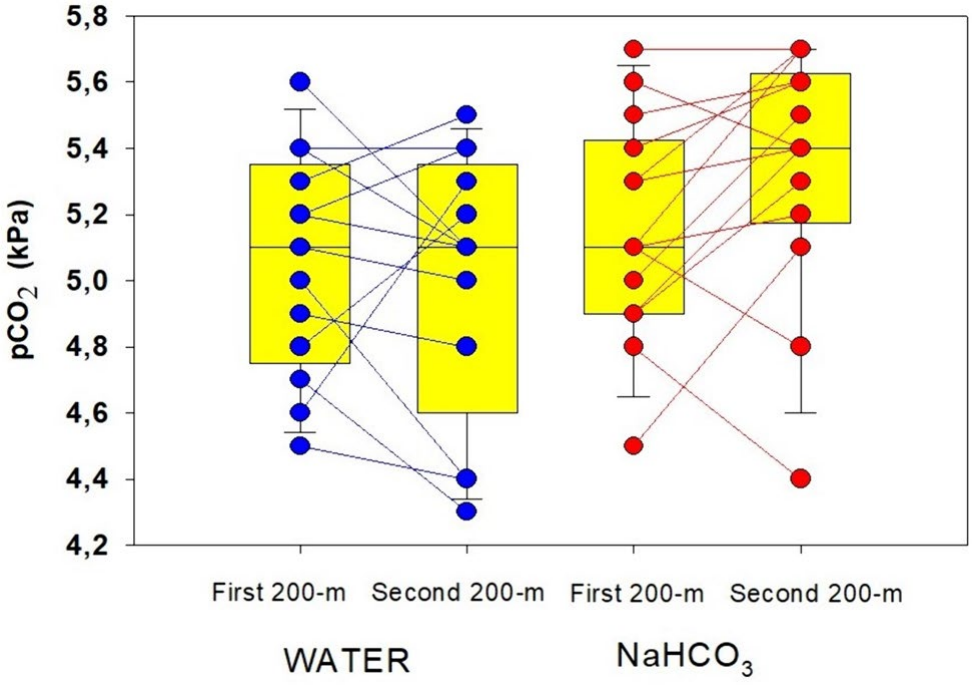
pCO_2_ values during the first minute of the first and second 200-m test with water ingestion and the first and second 200-m test with NaHCO_3_ ingestion. The results show a clear tendency for the pCO_2_ value to increase after NaHCO_3_ ingestion after the second 200-m test

**Fig. 3:**
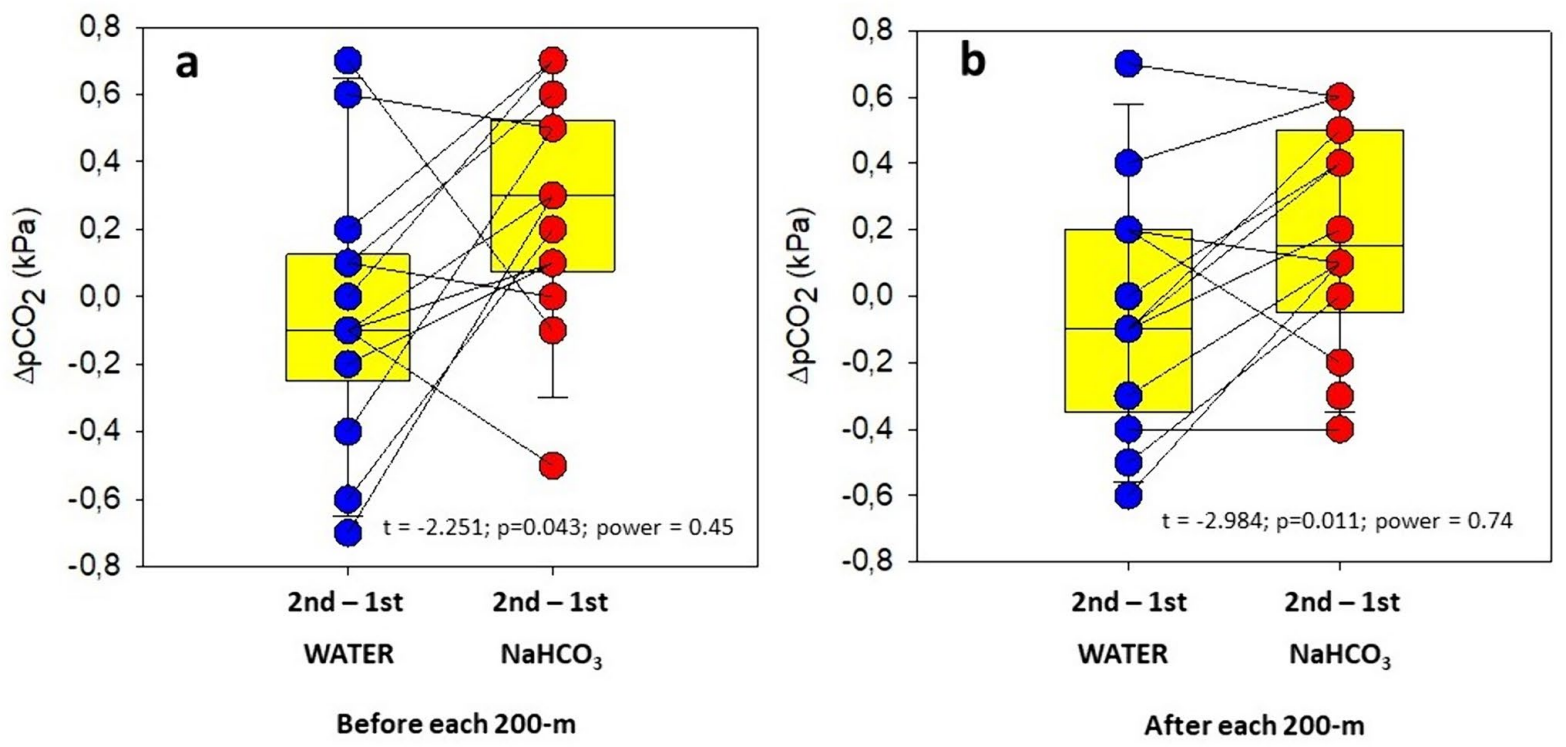
**a** ∆pCO_2_ for the 200-m test with water ingestion (∆pCO_2WATER_) compared to that for the 200-m tests with NaHCO_3_ ingestion (∆pCO_2NaHCO3_) before each test. ∆pCO_2_ was greater after the ingestion of NaHCO_3_ during the 1-h recovery period. **b** ∆pCO_2_ for the 200-m tests with water ingestion (∆pCO_2WATER_) compared to that for the 200-m tests with NaHCO_3_ ingestion (∆pCO_2NaHCO3_) during the first minute of recovery after each test. ∆pCO_2_ was greater after the previous NaHCO_3_ ingestion during the 1-h recovery period, likely because of the inability to increase ventilation during swimming, which could compensate for the “excess” CO_2_

In contrast to the other three trials, the ingestion of NaHCO_3_ before the second 200-m test had a significant effect on the values of the acid–base parameters at the first minute posttest (Table 2). Compared to the values of the other three 200-m tests, the pCO_2_ post value in the blood with NaHCO_3_ ingestion showed a clear tendency to increase by approximately 0.2 kPa (*F* = 1.056; *p* = 0.381; *η*^2^ = 0.088) (Table 2, Fig. 2). However, comparison of the differences in pCO_2_ between the two 200-m tests with water ingestion (∆pCO_2WATER_) with the differences in pCO_2_ post between the two 200-m tests with NaHCO_3_ ingestion (∆pCO_2NaHCO3_) revealed a significant change (*t* = − 2.984; *p* = 0.011; power = 0.74) (Fig. 3b). Compared to the values of the other three 200-m tests, two with water ingestion and one in with NaHCO_3_ ingestion, blood pH post 200-m test with NaHCO_3_ ingestion increased by approximately 0.11 (*F* = 5.255; *p* < 0.004; *η*^2^ = 0.323) (Table 2). The [${\text{HCO}}_{3}^{-}$] post value after the second 200-m test with NaHCO_3_ ingestion was approximately 5 mmol⋅L^−1^ (*F* = 18.55; *p* < 0.001; *η*^2^ = 0.628) (Table 2) higher than that with water ingestion and the first 200-m test. Similarly, compared to the other three 200-m tests, two with water ingestion and the first 200-m test with NaHCO_3_ ingestion, the highest BE post-values were observed (approximately 6 mmol L^−1^) (*F* = 10.85; p < 0.001; η^2^ = 0.497) (Table 2). Blood [LA^−^] post and SaO_2_ post as well as heart rate did not differ significantly throughout the experiment (Tables 2 and 3). In contrast, P_50_ post reached the lowest values after the second 200-m test with NaHCO_3_ ingestion (*F* = 6.266; *p* = 0.002; η^2^ = 0.410) (Table 3). Regardless of whether it was assessed for the whole body, the arm area or the legs, perceived exertion remained unchanged and was similar among the two reference trials and the two time trials (Table 4).

**Table 4:** Perceived exertion during the 200-m swimming tests

	WATER		NaHCO_3_	
	First 200 m	Second 200 m	First 200 m	Second 200 m_NaHCO3_
General exertion	8 ± 1	8 ± 1	8 ± 1	8 ± 1
Arm exertion	7 ± 1	7 ± 1	7 ± 1	7 ± 1
Leg exertion	8 ± 1	9 ± 1	8 ± 1	8 ± 1

WATER—denotes water-drinking conditions

NaHCO_3_—denotes NaHCO_3_-drinking conditions

## Discussion

Despite long-term investigations of the possible ergogenic effects of NaHCO_3_ ingestion during exercise, particularly swimming, the results are inconclusive. The aim of the present study was to determine the possible effects of NaHCO_3_ ingestion on performance in a 200-m front-crawl. Two maximal-effort 200-m front-crawl tests in which water was consumed during recovery were compared with two maximal-effort 200-m tests, in which the 0.3 g⋅kg^−1^ NaHCO_3_ solution was consumed during recovery between the two tests. The effect of NaHCO_3_ ingestion was associated with the following three main findings: (a) contrary to expectations, performance remained unchanged; (b) there was a strong alkalotic effect on blood acid–base balance with an increase in blood pH before and after swimming; and (c) there was a greater ∆pCO_2NaHCO3_ in the 200-m test with NaHCO_3_ ingestion compared to ∆pCO_2WATER_ in the 200-m tests with water ingestion, while absolute pCO_2_ only showed a clear tendency to increase after NaHCO_3_ ingestion. It is possible that a 1-h recovery interval is too short to have a significant effect on swimming performance after NaHCO_3_ ingestion despite the complex effects on blood acid–base balance. Indeed, the swimmers in the present study drank the NaHCO_3_ solution within 1 h of recovery (during the first 20–30 min). The shortest effective interval for between-trial NaHCO_3_ ingestion is suggested to be within 1 h after the first trial, which results in a recovery time longer than 1 h. The hydrogen carbonate buffering system in the blood may not contribute to the increase in swimming performance during the front-crawl, as acidosis during the swimming trial after NaHCO_3_ ingestion was weak due to the buffering effect. However, an increase in blood pCO_2_ appears to be an important factor under conditions of weak acidosis. The increase in pCO_2_ in the blood could force a swimmer to regulate their already restricted breathing to such an extent that hypercapnia could be avoided without severely fatiguing the respiratory muscles. However, this was not enough. To achieve this, the swimming speed must also be regulated and, in this case, remain the same. This is because reducing the swimming speed while maintaining the same ventilation can reduce the “excess” CO_2_ produced by the ${\text{HCO}}_{3}^{-}$ buffering system by slowing glycolysis and H^+^ release. However, this will increase the swim time, and the swimmer should consider whether this is an appropriate decision. Therefore, the effect of NaHCO_3_ ingestion on performance may not be as strong or similar if the acid–base status was previously increased.

One of the interesting interventions to improve swimming performance is to increase the capacity of the most important H^+^ buffer, hydrogen carbonate (Böning et al. 2007). It is possible that the ingestion of NaHCO_3_ solution (Burke 2013) partially compensates for exercise-induced metabolic acidosis in the blood (Bishop and Claudius 2005; Gao et al. 1988; Grgic 2022; Thomas et al. 2023; Montfoort et al. 2004; Zinner 2011). The alkalotic effect of this intervention is considered the main reason for the improvement in performance, especially in swimming (Gao et al. 1988; Pierce et al. 1992; Lindh et al. 2008; Joyce et al. 2012; Mero et al. 2013; Grgic and Mikulic 2021; Gough et al. 2023). In addition, the ability to accumulate more protons (H^+^ ions) and LA^−^ by starting with a higher resting [${\text{HCO}}_{3}^{-}$] and pH value and reaching the same levels at the end of exercise as those reached under conditions without NaHCO_3_ ingestion was also proposed as the main explanation for this phenomenon (Hollidge-Horvat et al. 2000; Siegler and Marshall 2015). However, this explanation has two major shortcomings: a) the ${\text{HCO}}_{3}^{-}$ buffer system produces relatively large amounts of CO_2_ that must be released from the blood compartment, and b) how blood alkalosis can increase muscle force and performance is not explained. The muscle cell membrane is relatively impermeable to ${\text{HCO}}_{3}^{-}$ (Hollidge-Horvat et al. 2000). In agreement with Stewart (1981), who calculated SIDs, the alkalotic reaction of the blood also influences the SID of the muscles by rearranging the concentrations of Na^++^, K^+^, Cl^−^, Ca^++^ and LA^−^ and thus also their electrochemical potential via the sarcolemma, leading to the conclusion that the intracellular SID and thus the redistribution of the electrochemical potentials of the ions can influence the excitation and contractility of muscle cells (Holidge–Horvat et al. 2000; Siegler and Marshall 2015; Sostaric et al. 2006; Stephens et al. 2002). Considering the changes in [H^+^] due to glycolysis and other sources (Robergs et al. 2004) and the changes in [Na^++^], [K^+^], [Cl^−^] and [LA^−^] due to depolarisation and ion transport through the sarcolemma, this part of the overall mechanism probably better explains how changes in ionic status affect muscle metabolism and performance (Lindinger et al. 1990; Raymer et al. 2004; Sostaric et al. 2006). In addition, there is a respiratory component of the ${\text{HCO}}_{3}^{-}$ buffer system that is required to regulate blood pCO_2_ levels (to prevent hypercapnia) and consequently the pH via ventilation (Siggaard-Anderson 1976). When NaHCO_3_ is ingested, an additional amount of CO_2_ is released through the buffering process, which also requires additional increased ventilation to prevent hypercapnia. This additional CO_2_ release is not a problem if ventilation is not restricted, e.g. during running when blood pCO_2_ falls below resting levels. However, our previous results suggest that this additional CO_2_ release may be a problem during front-crawl swimming, where pCO_2_ increases towards hypercapnic levels at the end of a 400-m front-crawl swim (Ušaj 1999). This phenomenon differs, for example, from that in maximal-effort kayaking, where the use of upper body muscles also dominates (Ušaj 1999). This difference is due to the limited increase in ventilation during the front-crawl and the interval at which the breath is held during an underwater turn every 50 m (Kapus et al. 2008); in the present study, this underwater turn occurred every 25 m. Although swimmers can increase ventilation through breathing frequency (Cardelli et al. 2000; Couto et al. 2014), this is still probably not sufficient to compensate for hypercapnic values without increasing the energy cost of the mechanical work of breathing. We, therefore, hypothesise not only that metabolic and electrolyte balance influences swimming performance following NaHCO_3_ ingestion but also that pCO_2_ levels severely limit 200-m front-crawl performance due to inadequate (costly) respiration. Since NaHCO_3_ ingestion helps to maintain muscle contraction but increased CO_2_ production can negatively impact performance, additional regulation of swimming velocity is needed. Finally, due to the unclear effects of NaHCO_3_ ingestion, it can be assumed that this intervention does not lead to an improvement in performance under all conditions, as in our study. McNaughton (2008) suggested that any ergogenic potential of NaHCO_3_ ingestion (alkalosis) depends on the physiological demands of sufficiently intense activity to induce performance-inhibiting metabolic acidosis. Since the blood pH only decreased to approximately 7.30 during the experiment in our study, we assumed that this limit had not yet been reached.

Even the increase in heart rate during exercise partly corresponds to increased oxygen delivery (Do_2_) and an increase in oxygen ingestion during exercise (Åstrand et al. 2004). Alkalosis reduces blood oxygen desaturation (Nielsen et al. 2002a, b), although we did not observe such a phenomenon. However, we observed a decrease in P_50_ during the second experimental trial with NaHCO_3_ supplementation (Chu et al. 2020) (Table 3). This observation can be explained by the Bohr effect, i.e. the easier binding of oxygen to haemoglobin (Sigaard–Andersen 1977). Whether alkalosis during swimming is related to oxygen consumption is unclear, and further observations are needed. However, the technical limitations of measuring gas exchange and ventilation during swimming without interfering with specific front-crawl-dependent breathing currently prevent such measurements from being made to a sufficient extent.

Limitations. As expected, ingestion of NaHCO_3_ before the second 200-m test increased blood alkalosis compared to the other three tests. However, contrary to expectations, the alkalosis achieved was also greater after the second test trial. The increased ${\text{HCO}}_{3}^{-}$ capacity was, therefore, not fully utilised. We have no explanation as to why the swimmers did not achieve the same acidosis when NaHCO_3_ was ingested as in the trials without this intervention. The timing of NaHCO_3_ ingestion could be one of the possible causes (Burke 2013; Grgic and Mikulic 2021).

Perhaps perceived effort, which did not differ among the different swimming trials in our study, is indicative of another cause of fatigue, suggesting that pCO_2_ levels are approaching the ‘danger zone’, at which point the swimmer is forced to maintain swimming velocity to prevent the negative effects of a combination of hypercapnia and metabolic acidosis. However, it is currently unclear whether this increase in pCO_2_ is accompanied by an increase in ventilation through an increase in breathing rate and consequently an increase in the mechanical work of the respiratory muscles. Alkalosis influences afferents III and IV, which partially inhibit the respiratory centres of the central nervous system (Siegler et al. 2015). The “central governor” theory (Noaks et al. 2003), which was additionally extended by the effort-based decision-making theory (Pageaux 2014), is ultimately responsible for decisions on how the swimmer regulates swimming velocity.

Conclusion. Several studies have suggested that NaHCO_3_ ingestion or infusion likely affects muscle performance via changes in electrolyte potential during relatively isolated muscle contractions, such as isokinetic knee extensions (Siegler and Marshall 2015), forearm muscle contractions (Raymer et al. 2004) and even finger contractions (Sostaric et al. 2006). However, the results were not as clear for physical activity, such as swimming. The intake of NaHCO_3_ did not always lead to an increase in swimming performance, as was also the case in this study. Because of these inconclusive results, our findings do not support the idea that swimming performance should depend solely on increased ${\text{HCO}}_{3}^{-}$ capacity. One possible reason for this difference is that the swimmers in our study did not achieve the same level of acidosis after the second 200-m test with NaHCO_3_ ingestion as they did after swimming in the 200-m test with water ingestion. In this case, the achieved alkalosis may not have such a strong influence on performance. The second reason could be that the pCO_2_ levels increased towards hypercapnic values, which additionally stimulates breathing. This is already restricted during the maximum front-crawl and cannot be increased by increasing the breathing rate, as the mechanical work of the respiratory muscles increases considerably. Thus, the increase in pCO_2_ becomes an important limiting factor that could have a negative impact on performance, in contrast to what would be expected with NaHCO_3_ ingestion. This novel aspect appears to be important for front-crawl stroke performance because of the complex relationship between swimming performance and the regulation of acid‒base status and ventilation. It can logically be applied to other swimming events and sports (rowing, cross–country skiing, etc.) where breathing must be coordinated with muscle contractions and upper limb movements.

## Data Availability

The datasets of this study are available from the corresponding author on the reasonable request.
